# Molecular Passports for Ex Situ Seed Donors: Enhanced Genetic Monitoring for Plant Conservation

**DOI:** 10.1111/mec.70324

**Published:** 2026-03-24

**Authors:** Jill A. Hamilton

**Affiliations:** ^1^ Department of Ecosystem Science and Management Pennsylvania State University University Park Pennsylvania USA

**Keywords:** biodiversity monitoring, conservation genetics, evolutionary potential, ex situ collections, genetic variation, Kunming–Montreal Global Biodiversity Framework

## Abstract

To maintain evolutionary potential, genetic diversity, the raw material upon which natural selection acts, must be conserved. As such, ex situ seed collections remain invaluable repositories of genetic diversity. However, questions remain regarding the degree to which collections preserve genetic diversity. A molecular passport, a genetic record sampled from an individual, geo‐referenced maternal seed donor, can complement traditional passport data maintained within an ex situ seed collection. The molecular passport provides an opportunity to standardise genetic monitoring of seed donors for species of concern and can identify gaps in preservation efforts, ensuring optimised allocation of resources associated with expanding conservation efforts. Molecular passports associated with seed donor populations enable tracking of demographic, neutral and non‐neutral evolutionary processes that have influenced standing genetic variation, can quantify genetic metrics for conservation decision‐making, and identify redundancies or confirm taxonomic identities critical to conservation efforts. Ultimately, the molecular passport can improve our ability to monitor and effectively preserve genetic variation for species at risk, turning a static resource into an actionable conservation decision‐making tool.

In a rapidly changing world, conservation requires the preservation of evolutionary potential to ensure species or populations have the capacity to adapt to changed conditions (Aitken et al. 2024). This capacity to adapt and evolve is tightly linked to the amount and distribution of standing genetic variation within and among populations. Thus, preservation of genetic variation, as the raw material upon which natural selection acts, remains essential for maintaining evolutionary potential. With growing emphasis to integrate genetic data into conservation policy and an explicit recommendation to incorporate genetic diversity metrics into the Kunming–Montreal Global Biodiversity Framework, new advances are being made to incorporate genetic data into species and population conservation management programs (Hoban et al. 2022; Robuchon et al. 2023; Schmidt et al. 2023). However, a key goal for species of concern remains to develop strategies that optimise preservation of evolutionary potential and the maintenance of genetic variation within and among populations (Hoban et al. 2024; Theissinger et al. 2023).

Ex situ seed collections, commonly maintained for agronomically important plant species, their wild relatives, and species of critical conservation concern, provide one pathway to conserve genetic diversity for species and populations outside of their native range. These collections aim to ensure genetically diverse, geo‐referenced germplasm are maintained as repositories, creating an invaluable resource that preserves individual plant species and population evolutionary potential (Di Santo and Hamilton 2021). For many agronomic species, these germplasm collections have undergone a digital shift in the post‐genomics era as preserved accessions for regionally and globally distributed species collections are sequenced and genetic variation within and across collections are assessed (Aubry 2023; Varshney et al. 2021). Several ambitious national and international initiatives have focussed on characterising genotypic diversity across agronomic landraces, bred varieties and wild relatives maintained in collection (McCouch et al. 2012; Milner et al. 2019; Schulthess et al. 2022; Wu et al. 2019). These projects have developed comprehensive approaches to incorporating genetic discovery from collections into genomics‐assisted breeding platforms optimising the use of gene bank allelic diversity for use in breeding climate‐smart cultivars with improved stress resilience or increased nutritional value (McCouch 2013; Varshney et al. 2021). The majority of crop species preserved as gene banks, including rice (Varshney et al. 2021), barley, wheat (Schulthess et al. 2022) and many more have aligned high‐throughput genotyping of diverse collections with phenotyping for desirable traits and apply genetic associations and genomic prediction to meet targeted outcomes. In this way, agricultural gene banks, which manage thousands of accessions, employ genetic discovery in a use‐oriented framework, where genomic data support plant breeding, trait discovery, accession curation and redundancy detection (Sansaloni et al. 2020; Yuan et al. 2024). Combined with advances in genotyping technologies, declining sequencing cost, the availability of reference genomes and centralised data repositories that facilitate genotype to phenotype inference, this ensures the genetic profiles for individual accessions across agronomic species collections provides an invaluable, long‐lasting pre‐breeding resource.

While significant advances have been made quantifying genetic diversity maintained in collections for agronomic species of economic interest, the integration of these practices remains limited for species of conservation concern. Ex situ seed collections often remain relatively limited when the primary mandate is to preserve a species at risk. Given this, there remain questions regarding the degree to which existing conservation collections capture the genetic variation needed to preserve species' evolutionary potential (Braasch et al. 2021; Bragg et al. 2022; Di Santo and Hamilton 2021; Gargiulo et al. 2025). This issue is particularly urgent for species of conservation concern for which individual or population extirpation can lead to irrevocable loss of genetic diversity (Des Roches et al. 2021; Gargiulo et al. 2025). There is a need to combine traditional provenance and taxonomic data associated with ex situ seed collections with genetic data from seed donors to evaluate the in situ demographic and evolutionary processes that have influenced genetic diversity for species at risk. Parental seed donors can be used to create the genetic record, or molecular passport, needed to quantify genetic variation preserved within and among populations maintained in plant collections (Figure 1). These data can then be efficiently used to inform species management and expanded conservation efforts that optimise preservation of evolutionary potential (Linan et al. 2024; Melton et al. 2025).

**FIGURE 1:**
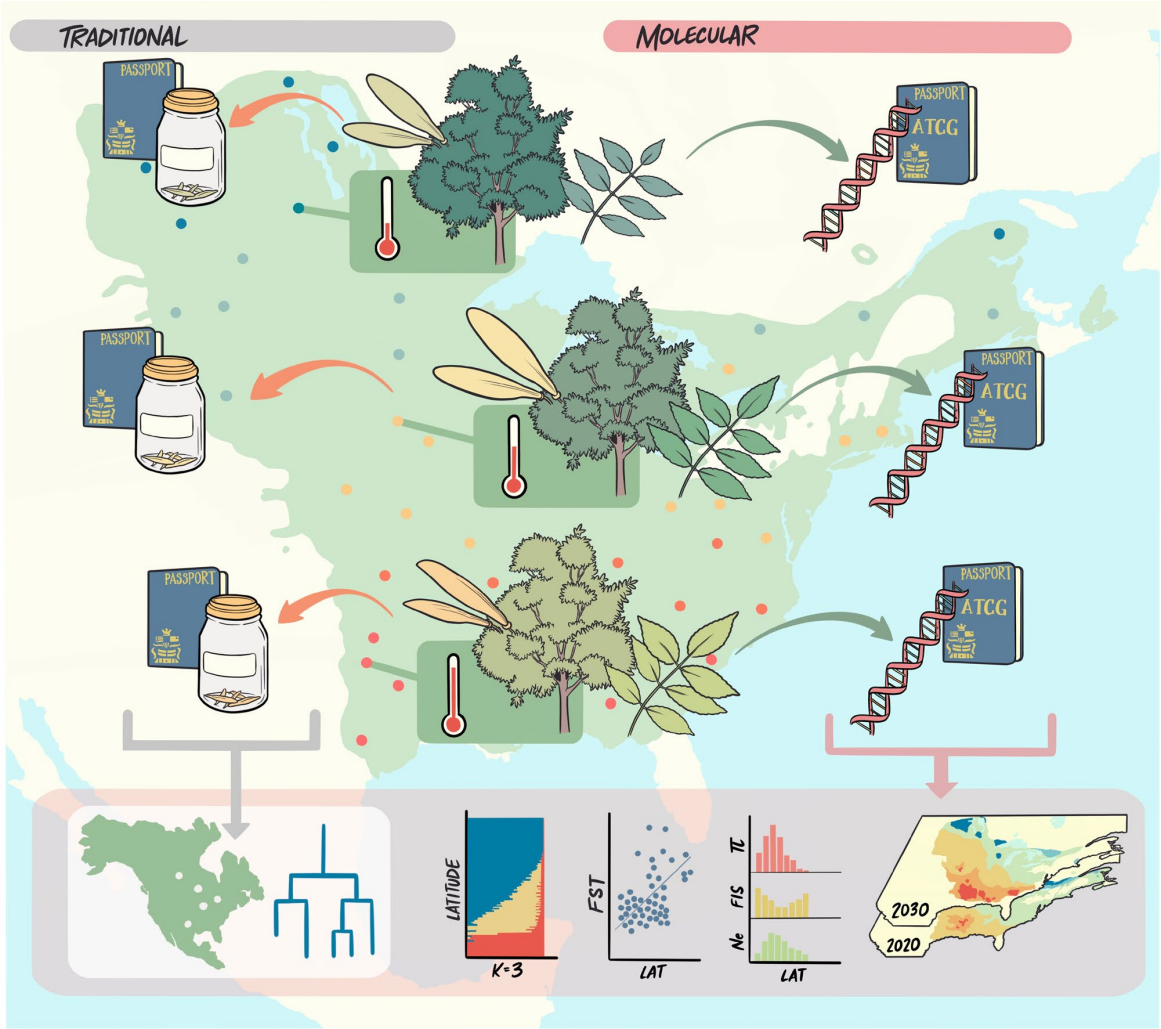
Traditional passport data associated with ex situ seed collections generally include provenance of origin and taxonomic identity, which may be leveraged in many ways, including resolution of phylogeny or phylogeography (grey bubble). However, to understand the evolutionary processes that have shaped genetic diversity within the seed collection there is a need to assess genetic diversity within and among the seed donors themselves. Using foliage to extract DNA from multiple seed donors per population and genotype individuals (minimum 4–6 individuals/population depending on sequencing approach) the resulting genetic data reflects the cumulative outcome of evolution in situ. Each sequenced seed donor generates a molecular passport, a geo‐referenced genetic record representing the standing genetic variation of individual seed donors sampled within and across populations. Collectively, molecular passports created from multiple seed donors per population can be used to characterise spatially explicit neutral and adaptive evolutionary processes that have shaped standing genetic variation. In addition to phylogeny and phylogeography (grey bubble), this may include assessments of population genetic structure, isolation‐by‐distance, range wide estimates of genetic diversity (*π*), estimates of relatedness (*F*_IS_), or effective populations size (*N*_e_), quantification of current and predicted future genotype‐environment associations, and more (pink bubble). Ultimately, the data preserved within the molecular passport can be directly applied to species management, restoration, and conservation policy decisions.

## 1 Genetic Monitoring With the Molecular Passport

**FIGURE 2:**
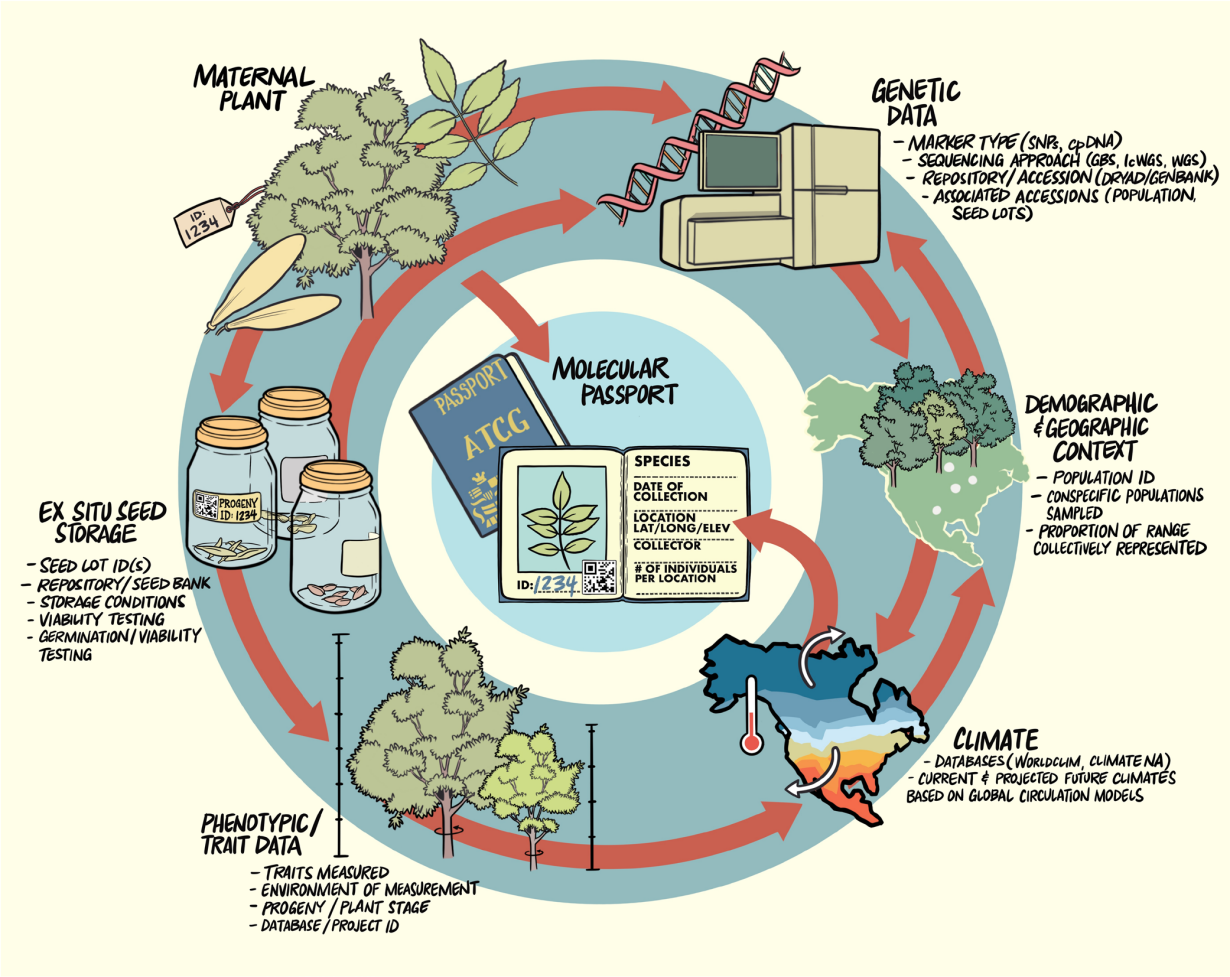
The molecular passport represents the genetic record sampled from a geo‐referenced seed donor to complement traditional data maintained alongside ex situ seed collections. Standardised provenance or origin data associated with the maternal plant ID will include species, date of collection, location of collection, collector, and number of individuals collected per location. Individual maternal plant identification will be maintained alongside progeny within the ex situ seed collections that may be stored under varying conditions, tested for viability or germination probability, or used to evaluate phenotypic traits across environmental conditions across seedling life history stages. For the maternal plant, standardised genetic data preserved in the molecular passport will include sequencing approach, genetic marker type, data repository, and associated genetic accessions, which may include conspecific population or progeny members if sequenced. The association with congener sampling within a species' distribution ensures genetic data can be associated with conspecifics to quantify the proportion of the range represented in collection. Finally, both the molecular passport and ex situ seeds in storage will benefit from association with climate of origin, calculated from latitude, longitude, and elevation of collection origin. This ensures climatic data associated with provenance origin during collection can be used to estimate future climatic projections using various global circulation models.

Traditionally passport data associated with ex situ seed collections describe the geographic origin or provenance of germplasm and taxonomy of the individual, largely based on morphology (Figure 1) (Mascher et al. 2019). More recently in many agronomically important species, the molecular passport has created a bio‐digital resource to complement, corroborate, and extend traditional passport information with genetic data and phenotypic data as a pre‐breeding resource (Mascher et al. 2024; Milner et al. 2019; Weise et al. 2020). The molecular passport, maintained at the level of the individual maternal seed donor, catalogues the natural genetic variation preserved within and among ex situ seed donors, effectively characterising the genetic variation represented within an accession's maternal lineage. Using DNA extracted from the foliage collected from the maternal ex situ seed donor, the molecular passport is generated as the genetic record for a single geo‐referenced, open‐pollinated seed donor whose progeny are preserved ex situ (Figure 1). The value of preserving the genetic profile of the seed donors alongside preservation of the seed itself is that, while both are products of natural selection, they represent different time points in the evolutionary and demographic history of the species. The genetic composition of the maternal seed donor reflects the cumulative effects of natural selection in the wild and the demographic factors that have influenced individual survival and reproduction within a genotype's native environment (Figure 2). This contrasts with the genetic composition represented within the ex situ seed collection, which represents a snapshot in time and space for an individual collection whose genetic variation may be shaped by anthropogenic influences associated with collection practice, curation or storage (Espeland et al. 2017). The difference between genetic variation maintained via natural selection for the seed producing plant in its native environment and variation potentially influenced by anthropogenic selection associated with seed collection and storage is subtle, yet fundamental to the conservation of genetic diversity. In this way, genetic data from multiple ex situ seed donors per population provides the opportunity to understand how natural selection and historical demographic processes have shaped standing genetic variation in the wild and recognises that the genetic variation preserved within ex situ collections may not fully capture the evolutionary dynamics influencing natural populations. The creation of these data can have direct application to the maintenance and growth of conservation collections, design of new or expansion of breeding programs and decision‐making related to species conservation and extinction risk.

Creating a digital repository of the molecular passports for the seed donors to complement ex situ seed collections is increasingly plausible for species at risk with new technological breakthroughs and the increasing affordability to create and curate next‐generation sequencing data for non‐model species (Figure 2) (Linan et al. 2024; Mascher et al. 2024; Melton et al. 2025). Genetic data preserved via molecular passports can characterise spatially explicit neutral and adaptive patterns of genetic variation for direct use in species management and conservation policies (Figure 1). The strength of the molecular passport is that it can be used to (i) evaluate microevolutionary processes that may have shaped standing genetic variation, (ii) model factors that may have impacted demographic change and (iii) quantify current and predict future genotype‐environment associations (Aitken et al. 2024; Bragg et al. 2022; Mascher et al. 2024, 2019; Melton et al. 2025; Rellstab et al. 2015) (Figure 1). Assessments of genetic variation may include individual estimates of expected heterozygosity (*H*_e_), genetic relatedness, or inbreeding (*F*_IS_) to indicate the distribution of genetic variation within and among populations represented ex situ. Kinship analyses within collections can serve as baseline data to inform the design of captive breeding programs (Linan et al. 2024). Population‐specific estimates of nucleotide diversity (*π*) or Watterson's *θ* can be used to evaluate how selective or demographic processes have contributed to variance in standing genetic variation following population founding (Braasch et al. 2021; Melton et al. 2025).

The Convention on Biological Diversity's Kunming‐Montreal Global Biodiversity Framework recommends the use of effective population size (*N*_e_) as a metric that incorporates an assessment of evolutionary processes underlying demographic change for monitoring species at risk of extinction (McLaughlin et al. 2025). Genetic data maintained from collections can also be used in demographic inference to model changes to effective population size over time while estimating the impacts that the rate and direction of gene flow may have had on population genetic structure (Bolte et al. 2024; Di Santo et al. 2022). Quantifying population genetic structure may identify genetic clusters underlying ecotypic variation that may inform conservation priorities. Together, these data may be used to compare how neutral and non‐neutral processes have contributed to the evolution of genetic differences among populations (Di Santo et al. 2022; Hamilton and Eckert 2007). Assessment of genotype–environment relationships may be used to infer the scale and extent of contemporary climate adaptation needed to inform assisted gene flow or climate‐assisted seed sourcing for restoration in the future if molecular passport data is available for populations across a species' range (Aitken and Whitlock 2013; Rellstab et al. 2015; Schiebelhut et al. 2023). Finally, the molecular passport may supplement incomplete records or resolve taxonomy where there are discrepancies in ancestry assignment associated with phenotype or provenance alone (Milner et al. 2019). In this way, genotypic characterisation via the molecular passport improves the reliability and accurate curation of traditional passport data (Singh et al. 2019).

Recommendations from the broader scientific community have advocated for the incorporation of genetic variation into conservation assessments and species management programs, including the International Union for Conservation of Nature (IUCN) red list assessments (Hoban et al. 2024; McLaughlin et al. 2025). The creation of the molecular passport for ex situ collections will directly aid in extending the use of genetic data for species at risk with direct applications to species management (Mastretta‐Yanes et al. 2024; McLaughlin et al. 2025; Rossetto et al. 2021). Ultimately, creation of molecular passports for species at risk will (i) standardise existing protocols needed to quantify and monitor genetic diversity (ii) identify gaps in current preservation efforts enabling coordination and optimisation of future collection efforts, (iii) identify redundancy in collections to ensure effective allocation of limited conservation resources, and (iv) confirm taxonomic status for individuals using genetic data (Mascher et al. 2024; Milner et al. 2019; Theissinger et al. 2023). These data will provide a foundational resource needed for comparative work across species with direct application to species conservation initiatives.

## 2 Considerations and Limitations of the Molecular Passport

While there are considerable advantages to the creation and broad use of the molecular passport, these data may still be limited by the size and quality of the collections themselves, which remain time consuming and expensive to implement (Di Santo and Hamilton 2021). Population genetic summary statistics, demographic modelling and genotype–environment associations will be impacted by variance in the number of individuals assayed per population, the number of populations sampled, and the proportion of the species' range that is covered by the collection. Recent simulation studies have suggested that a sufficiently large SNP data set can counteract potential negative effects associated with small sample size (Nazareno et al. 2017). Therefore, high‐throughput sequencing approaches, ddRAD‐Seq or whole genome sequencing will be most appropriate for creation of the molecular passport. Where there is a trade‐off between number of individuals assessed per population versus population diversity, recent studies suggest prioritising diverse populations as genetic diversity statistics are less sensitive to the number of individuals assessed per population (Aguirre‐Liguori et al. 2020). In addition, the sole assessment of the maternal seed donors in dioecious species may bias genetic structure. However, the use of open‐pollinated individuals should limit potential sex‐based biases. Finally, many seed banks are advocating tracking of ex situ collections by maternal lineage (van der Merwe et al. 2023). Maintaining a molecular passport for the seed donor alongside the tracked progeny will provide an opportunity to incorporate estimates of paternity and relatedness into evaluations of preserved genetic diversity. For plant species with mixed mating systems, this may identify regional variation in relatedness where supplemental sampling may be relevant.

Important considerations in generating the genomic data itself will include the reproducibility of data, the availability of a species‐specific or a conspecific reference genome, the choice of sequencing approach and the type of coverage required to explicitly address questions associated with conservation priorities (Hogg 2024). Sequencing technologies are rapidly evolving, which may limit the long‐term use or interoperability of genetic data. Recent recommendations from McLaughlin et al. (2025) emphasise the value of reduced representation or whole genome sequencing to genetic monitoring. Where whole‐genome resequencing is not possible for comparison across non‐model species, the creation of a standardised set of restriction enzymes for ddRAD sequencing, including one methylation‐sensitive enzyme to increase reproducibility across datasets, will enable effective comparison across species that may be critical to taxonomic identification (Melton et al. 2025). Comparisons performed in agronomic species indicate that there is a strong correlation between genetic profiles created via genotyping‐by‐sequencing and whole‐genome sequencing, suggesting that the relationships between reduced‐representation and whole‐genome matrices can be made and applied effectively (Milner et al. 2019; Schulthess et al. 2022). More recently, advancements in genetic profiling may consider data beyond nucleotide diversity and include structural polymorphisms that align to intermediate, reference or pangenome assemblies (Lind et al. 2025). Ultimately, ensuring the spatial and temporal maintenance of genetic records for the broader community will depend upon the degree to which raw data and metadata associated with sampling are curated and adhere to principles of FAIR data (findable, accessible, interoperable and reusable) (Crandall et al. 2023; Leigh et al. 2024). Newly generated genomic data should use standardised reporting, including individual genomic profiles for populations of maternal seed donors for each species, estimates of the proportion of the species' range sequenced and preserved ex situ, and documentation of the type of sequence data generated alongside number of genomic markers used (Figure 2; McLaughlin et al. 2025). Creating standard genomics workflows for the molecular passport across non‐models will be challenging, but it has the potential to revolutionise the depth of information accessible for species of conservation concern.

Finally, a major challenge to consider in the creation of molecular passports will be guidelines to ensure effective curation of bio‐digital data (Mascher et al. 2024). Creation and curation of open databases that represent archives for living seed collections and associated genetic data will need to be based on FAIR principles that ensure effective integration across traditional and molecular passports (Lind et al. 2025; Mascher et al. 2019). Some workflows that preserve links between genetic data and individual provenance data already exist for model and non‐model systems (i.e., CartograPlant; Lind et al. 2025). However, integration of traditional passport data associated with seed collection and individual maternal seed donor genetic data is needed (Figure 2). Achieving this integration will require the development and long‐term maintenance of relational databases capable of curating diverse data types. Modular, extendable frameworks that overlay existing platforms and emphasise the utility of shared data objects and flexibility as data types evolve will help ensure that these systems remain FAIR‐compliant and are scalable over time. With increasing globalisation of data and recommendations to integrate genetic data into conservation monitoring, this may also require standardised reporting across different platforms and will benefit from the creation of a unique digital object identifier (DOI) to curate seed and seed donor information that may be updated with expanded genetic data (Lind et al. 2025). Where genetic data are available within collections, they can be directly integrated into threat (re)assessments within and across species of concern (McLaughlin et al. 2025). Growing examples from agronomic species that reflect regional, national, and international partnerships offer pathways to build upon that will manage the co‐generation and sharing of technologies needed to preserve and leverage genetic data (Halewood et al. 2018). The GGI‐Gardens Project initiated alongside the Global Genome Biodiversity Network is piloting gold‐standard methods to not only voucher and preserve individuals within botanic gardens, but ensure a digital platform exists that can integrate genetic data alongside metadata maintained within collections to aid discovery and monitoring (Seberg et al. 2016). In this context, the value of the molecular passport will less likely be in maximising genomic resolution as it is for agronomic species, but more in ensuring that stored material captures and maintains genetic diversity and optimises representation across populations or lineages to provide a decision‐support tool for conservation and restoration initiatives (Cascini et al. 2025; Hogg 2024).

## 3 The Role of the Molecular Passport in Conservation Management and Policy

Conservative global assessments for plant species indicate 39% of all vascular plants are threatened with extinction, pointing towards an urgent need to expand existing monitoring efforts (Lughadha et al. 2020). The Global Strategy for Plant Conservation has explicit targets to preserve 70% of the genetic diversity for crops, wild relatives, and other socio‐economically valuable species globally (Target 9) and preserve 75% of known threatened plant species ex situ, ensuring 20% is available for recovery or restoration programs (Target 8) (Sharrock 2020). Ex situ seed collections remain a critical strategy needed to safeguard plant genetic diversity and ensure targets are met for conservation and restoration programs, but standardised monitoring to assess whether collections are meeting global standards are limited. Traditional passport data associated with ex situ collections can provide information on the number of individuals, populations, and proportion of a species' range that are maintained in collection, and where geographic and associated environmental data are available, these datatypes may be used as proxies to quantify diversity preserved (Di Santo and Hamilton 2021). However, with growing recognition that conservation collections lack sufficient intra‐specific genetic diversity needed to maintain evolutionary potential within recovery programs, genetic data provide the information needed to quantify whether collections harbour genetic diversity needed to meet global targets.

Where weak barriers to reproduction exist between sister species or where taxonomic identification is challenged by morphological assessment alone (Gargiulo et al. 2025), the molecular passport provides the record needed to accurately identify species needed to inform conservation goals (Cascini et al. 2025). The molecular passport can be used to directly inform Criterion D (current population size and restriction) and Criterion E (probability of extinction in the wild) as defined by the IUCN Red List (McLaughlin et al. 2025). These data will be an invaluable contribution to effective design and prioritisation of future efforts needed to ensure genetic targets are met for species at risk (Melton et al. 2025). There is also an opportunity to combine these data with comprehensive ongoing genomic biobanking activities within arboreta or botanic gardens (Seberg et al. 2016), provenance trials (Taylor et al. 2024; VanWallendael et al. 2022), and more to provide a foundation for estimating genetic diversity preserved ex situ. The Global Genome Biodiversity Network is creating means to aggregate genetic data and collection data to enable discovery and guide conservation (Seberg et al. 2016). Genetic monitoring of preserved collections will allow us to pinpoint regions across dynamic and changing landscapes to target additional conservation efforts, ensuring these data complement existing or ongoing threat assessments for species at risk.

Given the increasing need for proactive conservation in a rapidly changing world, creating molecular passports for seed donors preserved ex situ leverages increasingly accessible and affordable next‐generation sequencing data for species of conservation concern. Standardised genetic monitoring of seed donors for populations preserved ex situ can be used to quantify whether genetic targets are currently being met and can inform future policy and management efforts. These data may be used to tease apart the influence different demographic and evolutionary processes have had on the exchange and maintenance of genetic variation needed to ensure species' evolutionary potential is conserved (Theissinger et al. 2023). Normalising the routine collection of genetic data alongside ex situ collections bridges the research‐species management gap. Creation of the molecular passport transforms a static collection into an active asset for applied conservation, ensuring a preserved resource becomes actionable decision‐support tool.

## Funding

This work was supported by National Institute of Food and Agriculture (PEN04809, Accession 7003639) and The Schatz Center for Tree Molecular Genetics.

## Conflicts of Interest

The author declares no conflicts of interest.

## Data Availability

Data sharing not applicable to this article as no datasets were generated or analysed during the current study.
